# In-silico investigation of systematic missense mutations of middle east respiratory coronavirus spike protein

**DOI:** 10.3389/fmolb.2022.933553

**Published:** 2022-09-14

**Authors:** Raina Rhoades, Adebiyi Sobitan, Vidhyanand Mahase, Brhan Gebremedhin, Qiyi Tang, Danda Rawat, Hongbao Cao, Shaolei Teng

**Affiliations:** ^1^ Department of Biology, Howard University, Washington, DC, United States; ^2^ Howard University College of Medicine, Washington, DC, United States; ^3^ Department of Electrical Engineering and Computer Science, Howard University, Washington, DC, United States; ^4^ School of Systems Biology, George Mason University, Fairfax, VA, United States

**Keywords:** MERS-CoV, SARS-CoV-2, computational saturation mutagenesis, spike missense mutations, protein stability, S-DPP4 binding affinity

## Abstract

Middle East Respiratory Syndrome Coronavirus (MERS-CoV) causes severe pneumonia-like symptoms and is still pose a significant threat to global public health. A key component in the virulence of MERS-CoV is the Spike (S) protein, which binds with the host membrane receptor dipeptidyl peptidase 4 (DPP4). The goal of the present investigation is to examine the effects of missense mutations in the MERS-CoV S protein on protein stability and binding affinity with DPP4 to provide insight that is useful in developing vaccines to prevent coronavirus infection. We utilized a saturation mutagenesis approach to simulate all possible mutations in the MERS-CoV full-length S, S Receptor Binding Domain (RBD) and DPP4. We found the mutations in MERS-CoV S protein residues, G552, C503, C526, N468, G570, S532, S451, S419, S465, and S435, affect protein stability. We identified key residues, G538, E513, V555, S557, L506, L507, R511, M452, D537, and S454 in the S protein RBD region are important in the binding of MERS-CoV S protein to the DPP4 receptor. We investigated the effects of MERS-CoV S protein viral mutations on protein stability and binding affinity. In addition, we studied all DPP4 mutations and found the functional substitution R336T weakens both DPP4 protein stability and S-DPP4 binding affinity. We compared the S protein structures of MERS-CoV, SARS-CoV, and SARS-CoV-2 viruses and identified the residues like C526, C383, and N468 located in equivalent positions of these viruses have effects on S protein structure. These findings provide further information on how mutations in coronavirus S proteins effect protein function.

## Introduction

Emerging betacoronaviruses (β-CoVs) are a tremendous public health concern. The most recent one, SARS-CoV-2, has caused more than 6.2 million deaths worldwide (Adam, 2022). Other β-CoVs like SARS-CoV and MERS-CoV posed a threat to public health, with fatality rates of 9.4 and 34.4%, respectively (Petrosillo et al., 2020). MERS-CoV is endemic to Saudi Arabia and was isolated in 2012 from a sample of phlegm taken from a patient who died from pneumonia (Alkharsah et al., 2022). MERS-CoV, like SARS-CoV and SARS-CoV-2, is a member of the β-CoV family that contains a large group of enveloped RNA viruses and has zoonotic origin but can be transmitted from human to human. Like other coronaviruses, MERS-CoV can not only cause respiratory infections but also infects enteric and neurological systems. The original host for the MERS-CoV is a bat, the Egyptian Tomb Bat. The infection then was found its way to the reservoir source, known as the Arabian camel (Haagmans et al., 2014; Hu et al., 2015; Van Doremalen and Munster, 2015; Ramadan and Shaib, 2019). This zoonotic transmission from camels to humans continues in the Arabian Peninsula, with 19 cases of MERS-CoV reported in 2021 (European Center for Disease Prevention and Control, 2022). Although human to human transmission of MERS-CoV has been inefficient compared to other β-CoVs, there have been reports of co-infections of SARS-CoV-2 and MERS-CoV in Saudi Arabia and of a MERS-CoV related coronavirus in hedgehogs as well (Pomorska-Mól et al., 2022). These incidents highlight the possibility of the enhanced transmission or lethality of coronaviruses and create the conditions for a future pandemic (Pomorska-Mól et al., 2022; Uppalapati et al., 2022; Zhang et al., 2016).

A potential target of vaccine therapies for all three known β-CoVs is the Spike (S) glycoprotein. The S protein is responsible for helping the virus to gain entry to the cell contributing to the overall virulence of these β-CoVs. This protein is 1,353 amino acids in length, and the monomers assemble to form a homotrimer. The MERS-CoV S protein has 21 N-linked glycosylation sites (Wang et al., 2016). The S protein subunits are comprised of an S1 component and an S2 component. The S1 subunit is comprised of an N-terminal domain and C-domain, where the receptor-binding domain is found (Chan et al., 2015). The S1 RBD Core domain is made up of five anti-parallel β-sheets and two short helices within the connecting loops. The folding of the core subdomain is maintained by three disulfide bonds. The receptor binding subdomain of the RBD region is characterized by four β strands, which form an anti-parallel β-sheet. This receptor binding subdomain is found between the β4 and β9 strands of the core subdomain. A long loop connects the β6 and β7 strands traversing the antiparallel βsheet perpendicularly, and the loop is anchored by a disulfide bond connecting it with strand β5 of the receptor binding subdomain (Lu et al., 2013; Wang et al., 2013). The S2 subunit is comprised of a fusion peptide, two heptad repeat domains, a transmembrane domain, and a cytoplasmic domain (Walls et al., 2016; Yuan et al., 2017; Li et al., 2020). The heptad repeats when assembled form a fusion core that is inserted into the host cell membrane (Du et al., 2017). Membrane fusion and virus entry depend on the activity of both the S1 and S2 subunits. The S1 component is responsible for binding to a host cell receptor which initiates proteolytic cleavage at the boundary between the S1 and S2 protein regions. This proteolytic cleavage then causes a conformational change in the S2 component that results in the fusion peptide binding to the host cell membrane, and the two heptad repeat domains form a fusion core that facilitates the fusion of the viral and cell membranes and viral entry (L. Lu et al., 2014). Though the SARS-CoV, SARS-CoV-2, and MERS-CoV protein sequences may differ they each give rise to a protein that is approximately 180 kDa. The receptor utilized by the SARS-CoV and SARS-CoV-2 viruses is the angiotensin-converting enzyme 2 (ACE2) receptor located on the host cell’s membrane.

The MERS-CoV S protein binds to the dipeptidyl peptidase 4 (DPP4) receptor, also known as CD26. DPP4 is an enzyme that “cleaves dipeptides from hormones, chemokines, and cytokines.” It is expressed throughout the human respiratory system but is most abundant in the alveolar epithelium (Meyerholz et al., 2016). The preferential use of the DPP4 receptor was verified using transgenic mice expressing the human dpp4 gene (Van Doremalen and Munster 2015). While control mice failed to demonstrate viral replication following infection with MERS-CoV, transgenic mice expressing human DPP4 receptors demonstrated viral replication without the disease. However, after infection with the mouse-adapted MERS-CoV, transgenic mice demonstrated weight loss, decreased survival, and pulmonary pathology consistent with MERS-CoV infection (Li et al., 2015). Mice, ferrets, Syrian hamsters, cats, and dogs are among the only mammalian species that seem less susceptible to MERS-CoV infection (de Wit et al., 2013; Raj et al., 2013; Coleman et al., 2014; Van Doremalen and Munster, 2015; Kleine-Weber et al., 2020). A previous investigation found that MERS-CoV infection in humans is mediated by low-affinity interactions between the A domain of the S1 subunit and sialosides located on the DPP4 receptor (Park et al., 2019). Therefore, targeting the binding of MERS-CoV S protein and the DPP4 receptor may prove effective in preventing infection in humans. Based on structure modeling, the RBD region of the MERS-CoV S protein covers residues E382-C585 and S39-P766 of the extracellular portion of the DPP4 protein (Wang et al., 2013). The extracellular portion of the DPP4 protein consists of an N-terminal domain and eight β-propeller blades that are connected to a C-terminal α/β hydrolase domain. Each of the propeller blades consists of four anti-parallel β-strands. The S protein contains a binding motif that specifically targets the β4 and β5 propeller blades of the DPP4 (Lu et al., 2013a; Wang et al., 2013). The binding between the β4 and β5 propeller blades and the MERS-CoV S protein is driven primarily by hydrophilic residues, and the overall binding ranges from 10 to 20 nm (G. Lu et al., 2013; Barlan et al., 2014). This affinity is low in comparison to the affinity of the SARS-CoV S protein’s affinity for ACE, which is approximately ten times greater than that of the MERS-CoV RBD for DPP4 (Barlan et al., 2014).

Binding and subsequent fusion of the S protein is important for the virulence of coronaviruses. By targeting the binding of the S protein, the host cell receptor, one should theoretically be able to prevent or slow infection. The present study was designed to determine what effects missense mutations would have on the stability and binding affinity of the MERS-CoV S protein. Utilizing computational saturation mutagenesis, we generated all possible missense mutations in the MERS-CoV S and DPP4 proteins and evaluated the mutation effects on stability and binding affinity. Our results contribute to a greater understanding of how protein mutations affect the S protein function and may aid in the development of vaccines or other treatments.

## Materials and methods

### Structures

From Protein Data Bank (PDB) (https://www.rcsb.org/), we downloaded the MERS-CoV S ectodomain trimer (PDB ID: 5w9m) for analyzing the effects of mutations on full-length S protein stability. We chose this structure because it represents the prefusion spike and covers the full-length of the MERS-CoV spike protein sequence. The crystal structure of MERS-CoV complexed with human DPP4 (PDB ID: 4l72) was used to study the mutation effects on binding affinity and stability of the MERS-CoV S protein RBD and the DPP4 receptor, which the virus exploits to gain entry to the host (Berman et al., 2000). The SARS-CoV-2 S RBD (PDB ID: 6lzg) and SARS-CoV S protein RBD (PDB ID: 2ajf) were used for the comparison studies. We utilized PyMOL (http://www.pymol.org/) to carry out structure alignments and draw protein structural pictures.

### Mutations

We utilized a Perl script to generate a list of systematic mutations in each residue of the structures. These lists were utilized in to model these mutations in the complexed protein structure within the FoldX software (Schymkowitz et al., 2005). We also collected viral mutations for the MERS-CoV virus from the virus pathogen resource (ViPR) (Pickett et al., 2012). The disease-causing mutations in DPP4 were collected via the Human Gene Mutation Database (HGMD) (Stenson et al., 2003). We utilized the SNAP2 (Hecht et al., 2015) to determine the pathogenicity of mutations on the functionality of MERS-CoV S protein and DPP4.

### Energy calculations

We calculated the folding free energy change in the MERS-CoV full-length S protein and the binding free energy to assess the affinity between the MERS-CoV S protein and the DPP4 receptors. We utilized FoldX’s “RepairPDB” function to minimize the overall free folding energy of our structure. This repair structure was then utilized to perform systematic mutagenesis of amino acids and the folding free energy calculations. The “BuildModel” function within the FoldX program is used to generate mutagenized models of the repaired protein from a list of mutations provided by the user. The program then calculates the free energy change generated by the mutation by subtracting the ΔG of the control or repair structure from the ΔG of the mutagenized structure. The result is the stability change in folding free energy ΔΔG, where negative values are associated with increased stability, and positive values are associated with destabilization. The equation we used for protein stability is:

$$\Delta \Delta G=\Delta G_{\left(folding\right)}MUT-\Delta G_{\left(folding\right)}WT$$

A negative score (−0.5 to −2.5 or greater) indicates that the mutation in question has had a stabilizing effect on the overall protein structure. Scores between −0.5 and 0.5 are indicative of a neutral or negligible effect on protein stability. Finally, positive scores (0.5–2.5 or greater) indicate that the mutation has had a destabilizing effect on the overall protein structure.

Binding free energy changes were also calculated by the FoldX program using the “AnalyseComplex” function. This function unassembles each protein and assesses their individual energies, and then subtracts the individual energies from the energy of the protein complex to derive the ΔΔG_(binding)_. The energy change in the wildtype complex is then subtracted from the mutagenized protein complex to obtain the ΔΔΔG or change in binding free energy. Negative values signal an increase in binding affinity, whereas positive ΔΔΔG indicates a decrease in binding affinity. The binding energy was calculated using the following formula:

$$\Delta \Delta \Delta G=\Delta \Delta G_{\left(bindng\right)}MUT-\Delta \Delta G_{\left(binding\right)}WT$$

Similar to the protein stability scores, negative binding energy scores indicate increased binding affinity, while positive binding energy scores indicate decreased binding affinity.

## Results

### Protein stability changes in MERS-CoV S protein and RBD

**FIGURE 1:**
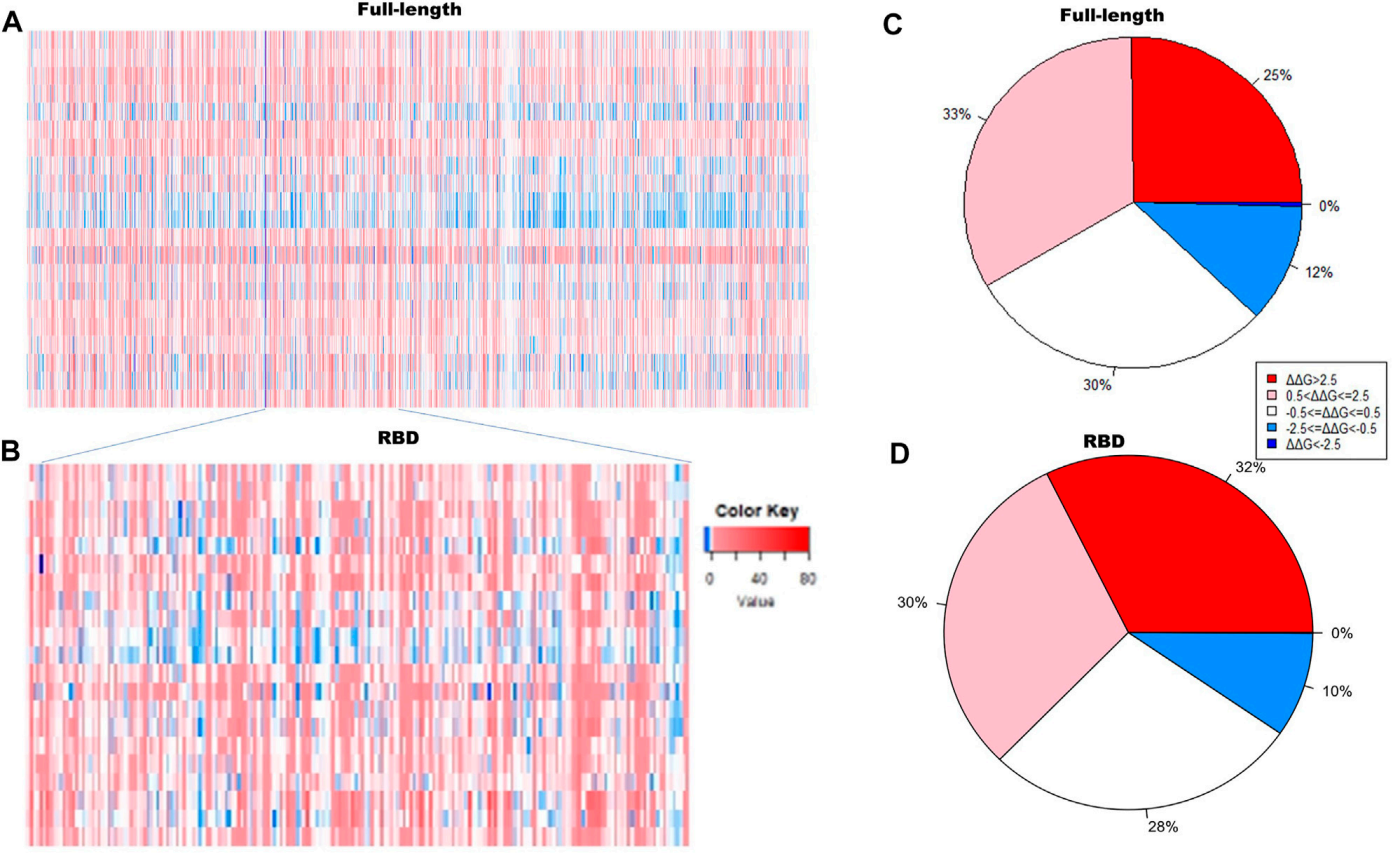
Stability heatmaps of the full-length **(A)** and the receptor-binding domain **(B)** MERS-CoV S protein. The pie charts represent the distribution of predictions of free folding energy following mutagenesis of the **(C)** full-length MERS-CoV S protein and the **(D)** receptor-binding domain (RBD) of the MERS-CoV S protein.

We generated 22,591 non-redundant mutations in the full-length MERS-CoV S protein and 3,876 non-redundant mutations in the MERS-CoV S protein RBD region. Figure 1 displays the stability heatmaps (Figures 1A,B) and the percentages of highly destabilizing to highly stabilizing mutations (Figures 1C,D) with respect to the full-length and RBD structures. A similar pattern of free folding energy predictions was found following mutagenesis of the full-length S protein. Most of the mutations generated resulted in moderately to highly destabilizing effects on the full-length S protein structure, approximately 33 and 25%, respectively (Figure 1C.). About 20% of mutations generated in the full-length MERS-CoV S protein were moderately stabilizing, and 30% of mutations generated were predicted to have a neutral effect. Within the MERS-CoV S protein RBD region (Figure 1D.), 32% of mutations that were generated were expected to have a highly destabilizing effect on protein structure, and 30% were expected to be moderately destabilizing. Of the remaining mutations generated in the MERS-CoV S protein RBD, 28% of the mutations generated were predicted to be neutral, and 10% were found to be moderately stabilizing.

### Protein stability changes in S protein RBD subregions

**FIGURE 2:**
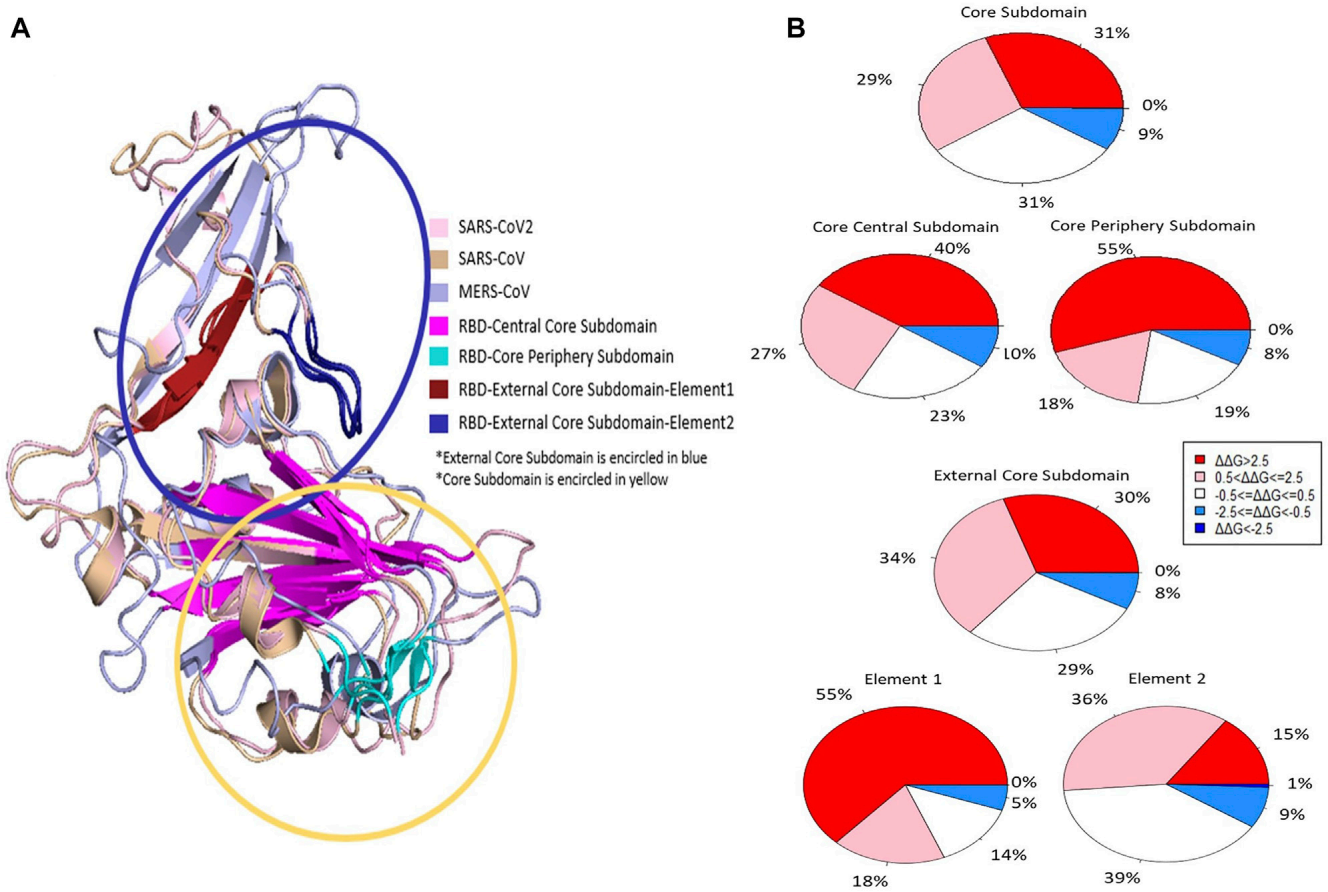
Structure of the RBD regions of SARS-CoV-2, SARS-CoV, and MERS-CoV S proteins **(A)**. The percentages of highly destabilizing, moderately destabilizing, neutral, and moderately stabilizing mutations found within the core and external core subdomains are depicted in **(B)**.

The RBD region can be divided into core and external core subdomains (Figure 2A.). The external core is responsible for receptor binding. The core can be further divided into the central core and peripheral core. The central core is composed of five β-strands. This conserved β-sheet was observed in S protein RBD regions of SARS-CoV as well as Bat coronavirus HKU4 and HKU9 (Huang et al., 2016). Helps to stabilize the C and N terminals in the correct formation. We evaluated the core and external subdomains based on their stability changes resulting from mutagenesis. The external core subdomain and core subdomains are very similar in terms of the relative proportion of stabilizing and destabilizing mutations. We decided to investigate the components of each subdomain to determine if there are any regions of interest. Mutations generated within the external core subdomain were mostly predicted to be destabilizing. Approximately 30% of mutations were found to be highly destabilizing, while approximately 34% were found to be moderately destabilizing. Around 29% of the mutations were found to be neutral, and 8% were found to be moderately stabilizing to the MERS-CoV RBD region. Within the external core are two small regions that anchor the external core to the core subdomain known as element one and element 2 (residues 497-503 and residues 517-524, respectively) (Figure 2B.) (Huang et al., 2016). We evaluated the effect of mutagenesis on the stability of element 1, element 2, and the remaining external core sequence positions. Though element one is relatively small, 55% of mutations generated in this region were found to be highly destabilizing, 18% of mutations were found to be moderately destabilizing. The remaining 19% were found to be neutral, and 8% were found to be moderately stabilizing. The distribution of predicted effects on protein stability is different for element 2. Of the mutations generated in element 2, 15% were found to be highly destabilizing, 36% were moderately destabilizing, 39% were found to be neutral, 9% were found to be moderately stabilizing, and 1% were found to be highly stabilizing. We then investigated the components of the core subdomain. There we found that 31% of the mutations we generated were highly destabilizing, 29% were moderately stabilizing, 31% were neutral, and 9% were found to be moderately stabilizing (Figure 2B).

Three disulfide bridges help to stabilize the core subdomain; C383 to C407, C425 to C478, and C437 to C585 (Lu et al., 2013). If mutagenesis occurs in any of these six positions results in mean decreased stability change. The greatest change in mean destabilization is found at position 383, located at the beginning of the RBD region (mean ΔΔG = 8.13665 kcal/mol). None of these residues are predicted to have any effect on the binding affinity between MERS-CoV S protein RBD and DPP4. Of the mutations we generated in the core subdomain, most of the mutations were predicted to cause destabilization of the MERS-CoV S protein RBD were found in the core-periphery subdomain.

### Key positions affecting S protein RBD protein stability

**FIGURE 3:**
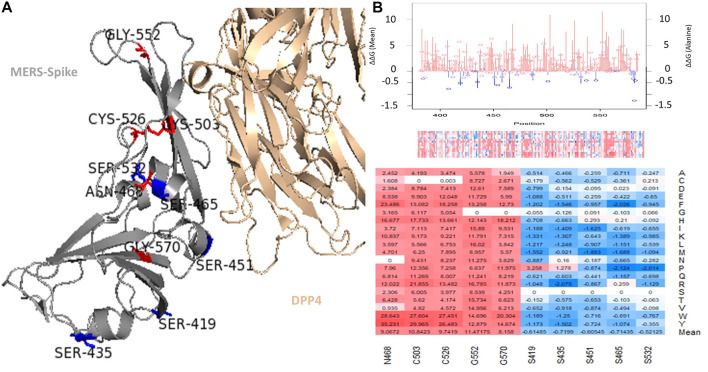
Top mutations in the MERS-CoV S RBD. **(A)** Illustrates the top mutations in the MERS-CoV S RBD, which is shown in complex with the DPP4 receptor. **(B)** Depicts the energy landscape of the MERS-CoV S protein and top mutations in the MERS-CoV. The line graph depicts the mean ΔΔG, with red lines indicating destabilization and blue lines indicating stabilization. The bubbles indicate the predicted ΔΔG of alanine mutations. The MERS-CoV S protein heatmap is shown beneath the line graph. The stability heatmap is directly beneath the line graph. The bottom of panel B displays the top positions with respect to mean stabilization and destabilization.

The top positions in the MERS-CoV S protein RBD with respect to mean stability are displayed in the stability heatmap and line chart in Figure 3. The top five destabilizing (N468, C503, C526, G552, and G570) and top five stabilizing (S419, S435, S451, S465, and S532) positions based on mean stability are displayed in Figure 3B. Three of the five top positions, N468, C503, and C526, are associated with a decrease in stability and are contacting residues between MERS-CoV S protein and DPP4 (mean ΔΔG = 9.06, 10.84, and 9.74 kcal/mol). Two positions that are not associated with DPP4 binding directly are also predicted to be highly destabilizing on the overall structure of the MERS-CoV S RBD: C552 and G570 (mean ΔΔG = 11.47 and 8.15 kcal/mol). The mutations in several serine (S) residues were found to have an overall stabilizing effect on the RBD region: S419, S435, S451, S465, and S532 (mean ΔΔG = -0.61, -0.72, -0.61, -0.71, -0.52 kcal/mol).

### Binding affinity changes in S RBD

**FIGURE 4:**
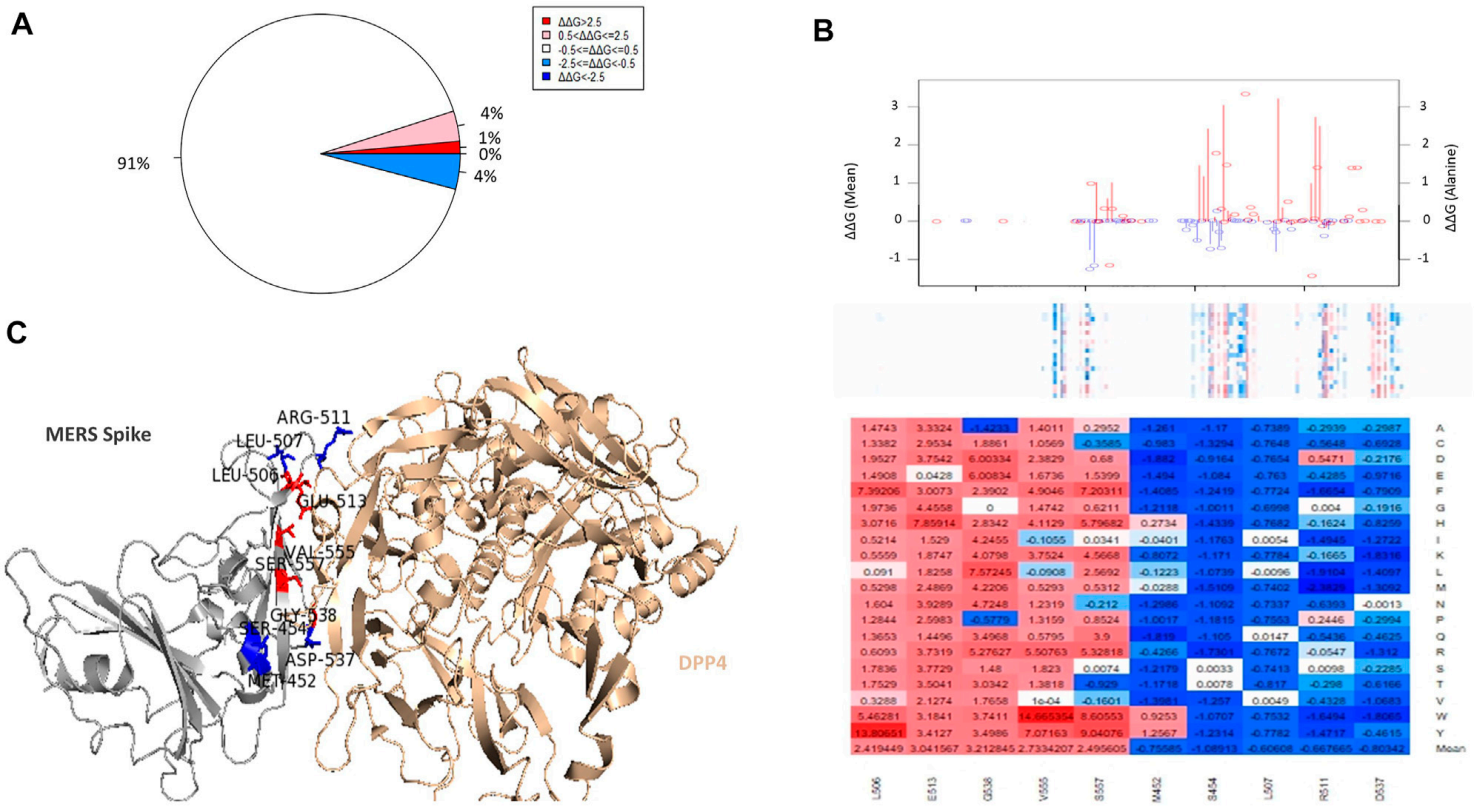
**(A)** Percentages of ΔΔΔG values that represent increased or decreased binding affinity in the MERS-CoV S RBD. **(B)** The energy landscape with respect to binding affinity. The affinity heatmap is depicted below. And the chart at the bottom displays the ΔΔΔG values for the top stabilizing and destabilizing residues. **(C)** Picture displays the position in the MERS-CoV S RBD with respect to binding affinity.

In terms of the effect of mutagenesis on the binding affinity between MERS-CoV S protein RBD and the DPP4 receptor, there are more interacting residues within the external core subdomain, several of which are associated with a mean decrease in binding affinity. However, mutagenesis is generally predicted to have a neutral effect on affinity, as percentages of the highly destabilizing, moderately destabilizing, neutral, and moderately stabilizing mutations found in the external core region are: 1, 4, 91, and 4%, respectively (Figure 4A.). Several of the top positions associated with a mean increase or decrease in binding affinity to the DPP4 receptor are shown in Figure 4B: L506, E513, G538, V555, S557; and M452, S454, L507, R511, and D537. Several of these positions are also located at the interface between the MERS-CoV S protein RBD and the DPP4 receptor: L506, E513, D537, G538, V555, R511, and D537 (Figure 4C.).

**FIGURE 5:**
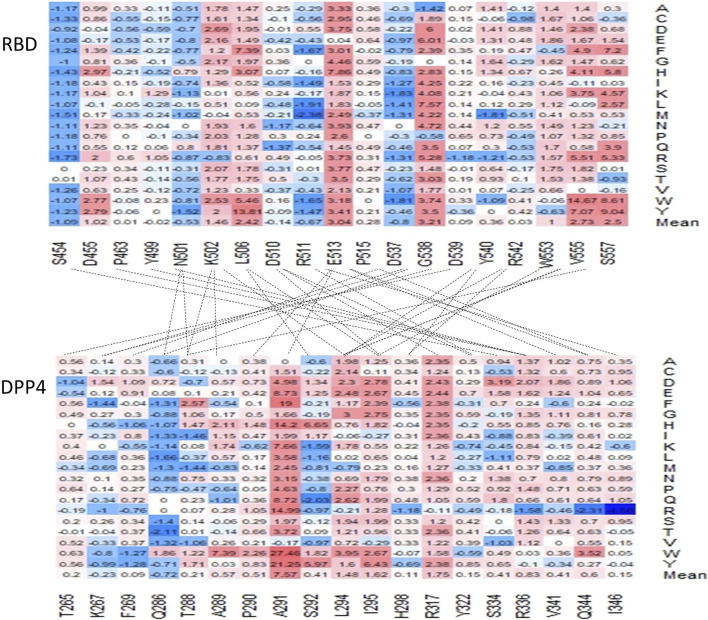
Charts of the predicted ΔΔΔG of residues predicted to interact within the interface of the MERS-CoV S protein and the DPP4 receptor. The lines indicate which residues are predicted to contact another in the two proteins.

We also evaluated the effect of mutations on the contact residues predicted by the Protein Contacts Atlas (Figure 5) (Kayikci et al., 2018). Mutagenesis in several of these positions, namely D455, K502, L506, E513, W553, V555, and S557, are expected to have a moderate to highly destabilizing mean effect on the overall protein affinity for the DPP4 receptor, all of which were predicted to have mean stability changes that are greater than or equal to 1.0 kcal/mol (Figure 5).

### Viral variants in MERS-CoV S protein RBD

**FIGURE 6:**
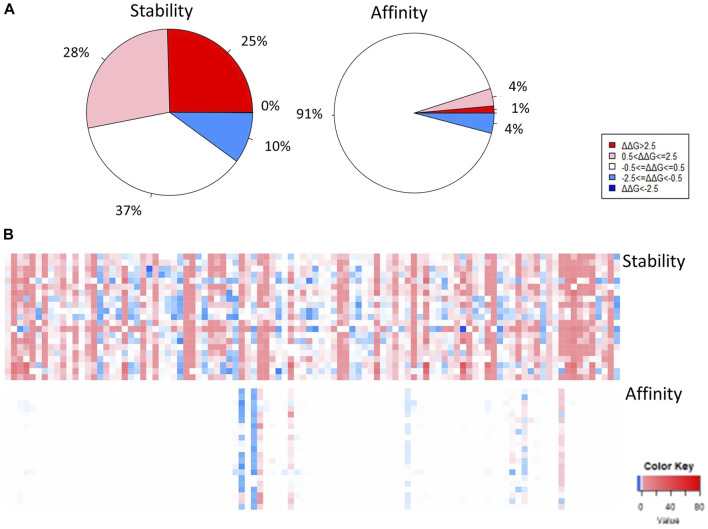
Stability and binding affinity of positions associated with reports of viral variants obtained from ViPR, displayed in pie charts **(A)** and in heatmaps **(B)**.

We collected viral variants collected from the Viral Pathogen Resource (ViPR) relevant to the MERS-CoV S protein RBD. The effect of variation on protein stability and affinity are displayed for each position in the MERS-CoV S protein RBD, which corresponds to a variant reported in the ViPR database. The Viral Pathogen Resource currently lists 1750 viral variants for the full-length MERS-CoV S protein RBD. Of the variants, we acquired 104 that were located within the MERS-CoV S protein RBD. Their effects on protein stability, binding affinity and mutation pathogenicity are summarized in Supplementary Table S1. Most positions in the RBD region were predicted to be moderately to highly destabilizing (28% moderately destabilizing and 25% highly destabilizing). The remaining mutations generated in positions relevant to viral variants were found to be stabilizing or neutral (10% moderately stabilizing and 37% neutral) (Figure 6A). A heatmap of the positions of viral variants reported in ViPR can be found in Figure 6B. The top stabilizing viral variants within the MERS-CoV S protein RBD were: S429D, Q471M, N487D, S451R, T424I (ΔΔG = -2.854, -1.700, -1.296, -0.867, -0.657 kcal/mol, respectively) (Table 1). The top destabilizing viral variants were G570H, L506F, F418S, Y438S, F571T (ΔΔG = 18.212, 10.775,6.452, 5.297, 4.830 kcal/mol, respectively) (Table 1).

**TABLE 1:** The effects of top viral variants on protein stability, binding affinity and mutation pathogenicity.

Viral variant	FoldX		SNAP2		
	ΔΔG	ΔΔΔG	Effect	Score	Accuracy (%)
F418S	6.452	0	effect	50	75
T424G	1.189	0	effect	50	75
T424I	-0.657	-1.00E-04	effect	44	71
S429D	-2.854	0	neutral	-5	53
Y438S	5.297	0	effect	78	85
S451R	-0.867	-0.3479	neutral	-10	53
M452L	0.31	-0.1223	effect	1	53
K453E	1.009	-0.0597	effect	43	71
D455N	1.702	1.2273	neutral	-35	66
S460G	-0.128	0.3141	effect	52	75
Q471M	-1.7	0	effect	10	59
N487D	-1.296	0	neutral	-1	53
L506F	10.775	7.3921	effect	75	85
T533Y	0.22	-0.0409	effect	52	75
D539N	0.453	0.4442	neutral	-14	57
Y541L	1.858	-0.0634	effect	48	71
W553R	3.235	1.5675	effect	84	91
F571T	4.83	0	effect	74	85

Of the mutations generated in positions corresponding to viral variants in the MERS-CoV S protein RBD region, 91% were found to be neutral, 1% were found to decrease binding affinity significantly, 4% were found to cause a moderate decrease in protein affinity, and 4% were found to cause a moderate increase in protein affinity (Figure 6A). The top mutations in terms of increasing protein affinity are S451R, M452L, Y541L, K453E, T533Y (ΔΔΔG = -0.348, -0.122, -0.634, -0.059, -0.040 kcal/mol, respectively) (Table 1). The top mutations in terms of decreasing protein affinity are L506F, W553R, D455N, D539N, and S460G (ΔΔΔG = 7.392, 1.567, 1.227, 0.444, 0.314 kcal/mol, respectively) (Table 1).

However, it seems that even small effects on the protein stability and binding affinity of the MERS-CoV S protein RBD might be able to have a significant effect on viral entry. Kim and others reported two variants in the MERS-CoV S protein RBD, I529T and D510, that reportedly had reduced affinity for the DPP4 receptor (Kim et al., 2016). D510 is associated with the interface of DPP4 in the MERS-CoV S protein RBD. Results of our analysis predict that a mutation from aspartate to glycine at this position is predicted to have a relatively neutral effect on both protein stability and protein affinity for the DPP4 (ΔΔG = 0.44, ΔΔΔG = 0.3629 kcal/mol). The I529 to T mutation is predicted to cause a significant change in protein stability and a neutral change in protein affinity (ΔΔG = 3.124, ΔΔΔG = 0.4841 kcal/mol). However, a previous investigation reported that EC50 for viral strains with D510G and I529T mutations increased by 2-fold and 20-fold (Kim et al., 2016).

### Binding affinity changes in DPP4

**FIGURE 7:**
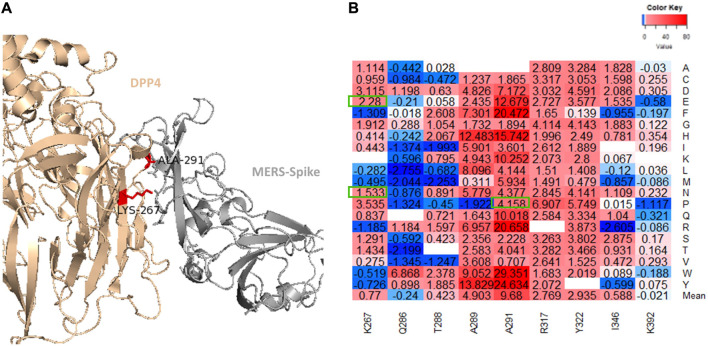
**(A)** Illustration of the MERS-CoV S protein and DPP4 interface with the K267 and A291 residues displayed. **(B)** The binding affinity heatmap and ΔΔΔG values for mutations generated in each position investigated by Kleine et al. (2020).

Figure 7 displays the residues in the DPP4 receptor that are predicted to interact with the MERS-CoV S protein based on the protein contacts atlas. A recent paper investigating 14 naturally occurring variants in DPP4 found that three variants: K267E, K267N, and A291P located in binding interface (Figure 7A) can reduce the binding affinity of the MERS-CoV S protein based on the results of flow cytometry and co-immunoprecipitation (Kleine-Weber et al., 2020). The authors also reported that all four variants reduced viral entry into the host’s cells. However, the K267E and A291P mutations were associated with reduced viral replication (Kleine-Weber et al., 2020). As shown in Figure 7B, K267E, K267N, and A291P can weaken the protein-protein interactions between DPP4 and MERS-CoV S protein RBD. Other key residues, including Q286, A289, L294, I295, R317, and R336, are predicted to have moderate effects on the protein binding affinity of the DPP4 receptor.

### R336T substitution in DPP4

Previous investigations of the permissiveness of model organism cells to MERS-CoV infection have shown that a relatively small number of mammals are less susceptible to infection. Alignment and comparison of human, mouse, and Syrian hamsters’ DPP4 sequences reveals a high degree of conservation. However, in mice and Syrian hamsters, there is an R336T substitution that removes a positive charge (Van Doremalen and Munster 2015). This substitution also introduces a glycosylation site in these rodents. The R336T position contacts D455, P463, and Y499 residues in the MERS-CoV S protein RBD (Figure 7). All three are located in the external core subdomain. The R336 to T mutation increases the distance between itself and these residues. Evaluating the impact of this substitution on protein stability and binding affinity, we find that the R336T substitution weakens both protein stability and affinity (ΔΔG = 3.34 kcal/mol and ΔΔΔG = 1.25 kcal/mol). The majority of mutations in the P463 and Y499 residues cause neutral changes in the binding affinity between MERS-CoV S protein and DPP4, although if lysine or arginine is introduced in position 499, the result is a moderate change in affinity (ΔΔΔG values are between 1.0486 and 1.285 kcal/mol). The mean predicted effect of missense mutations in position 455 is a moderate decrease in affinity (mean ΔΔΔG = 1.018 kcal/mol), although histidine, tryptophan, and tyrosine substitutions are likely to be highly destabilizing to the MERS-CoV S protein DPP4 complex (ΔΔΔG >2.7 kcal/mol).

### Comparison of the S protein RBD regions of MERS-CoV, SARS-CoV, and SARS-CoV-2

To compare the S protein RBD regions of MERS-CoV, SARS-CoV, and SARS-CoV-2, we performed multiple sequence alignment of the S protein RBD regions of the MERS, SARS, and SARS-CoV-2 using Clustal Omega (Supplementary Figure S1). We performed a multiple sequence alignment using Clustal Omega and found the percent identity between the MERS-CoV and the SARS-CoV and SARS-CoV-2 RBD regions are 17.99 and 19.10%, respectively. The percent identity between SARS-CoV and SARS-CoV-2 is 72.88%. Although the sequences of the MERS-CoV S protein and the SARS-CoVand SARS-CoV-2 S proteins differ in terms of their sequence, they are very similar to one another in terms of their overall structure (Figure 1). The RMSD values of the structural alignment between the MERS-CoVS protein RBD and those of the SARS-CoV and SARS-CoV-2 S protein RBD regions are 1.834 Å (204–195 residues) and 1.818 Å (204–174 residues), respectively.

Of the top position in terms of mean free folding energy change after mutagenesis in the MERS-CoV S protein RBD structure, seven map precisely to positions containing the exact same original amino acid in the SARS-CoV and SARS-CoV-2 S protein RBD structures, three within the external core, and three within the central core. Within the external core, correlates of C526, G552, and G558 within the MERS-CoV S protein RBD structure are all highly destabilizing (ΔΔG >3 kcal/mol). The C526 residue and its correlates in SARS-CoV and SARS-CoV-2, cysteines C467 and C480, form a disulfide bridge with cysteines within the external core subdomain. A comparison of the two anchoring portions of elements 1 & two and the equivalent positions in the SARS-CoV and SARS-CoV-2 proteins demonstrated several residues that result in an overall decrease in protein stability. However, these positions vary with respect to their effect on protein binding affinity.

Within the central core, correlates of C383, C407, and G462 within the MERS-CoV S protein RBD structure are moderately to highly destabilizing (mean ΔΔG >1.19 kcal/mol). There is a difference, however, in the correlates of position N468. In S protein RBD regions of MERS-CoV SARS-CoV and SARS-CoV-2, the mean free folding energy change for N468, N422, and N409 is predicted to be highly destabilizing, and each is greater than 6 kcal. However, in terms of their effect on binding affinity, mutations in this residue in MERS-CoV S protein RBD are expected to decrease binding affinity, while mutations in the equivalent position in SARS-CoV and SARS-CoV-2 S protein RBD regions are predicted to have a neutral effect on protein binding.

There are four disulfide bridges within the RBDs of the MERS-CoV, SARS-CoV, and SARS-CoV-2 S proteins. Three disulfide bridges stabilize the central core region, while the remaining disulfide bridge is found in the distal receptor binding domain. Mutations in the central core and external core subdomains affect the protein stability but have a neutral effect on the affinity between the S protein and its target host receptor.

Both the overall MERS-CoV and SARS- CoV S protein RBD external subdomains exhibit similar percentages with respect to mutations resulting in increasing or decreasing stability. However, 19% more mutations in the MERS-CoV S protein external core subdomain element one were predicted to be highly destabilizing than in the SARS-CoVS protein external core subdomain. I addition, a greater percentage of mutations in the element two component of the SARS-CoV S protein external core subdomain are predicted to be moderately destabilizing relative to the MERS-CoV S protein element two component (59 and 35%, respectively).

**FIGURE 8:**
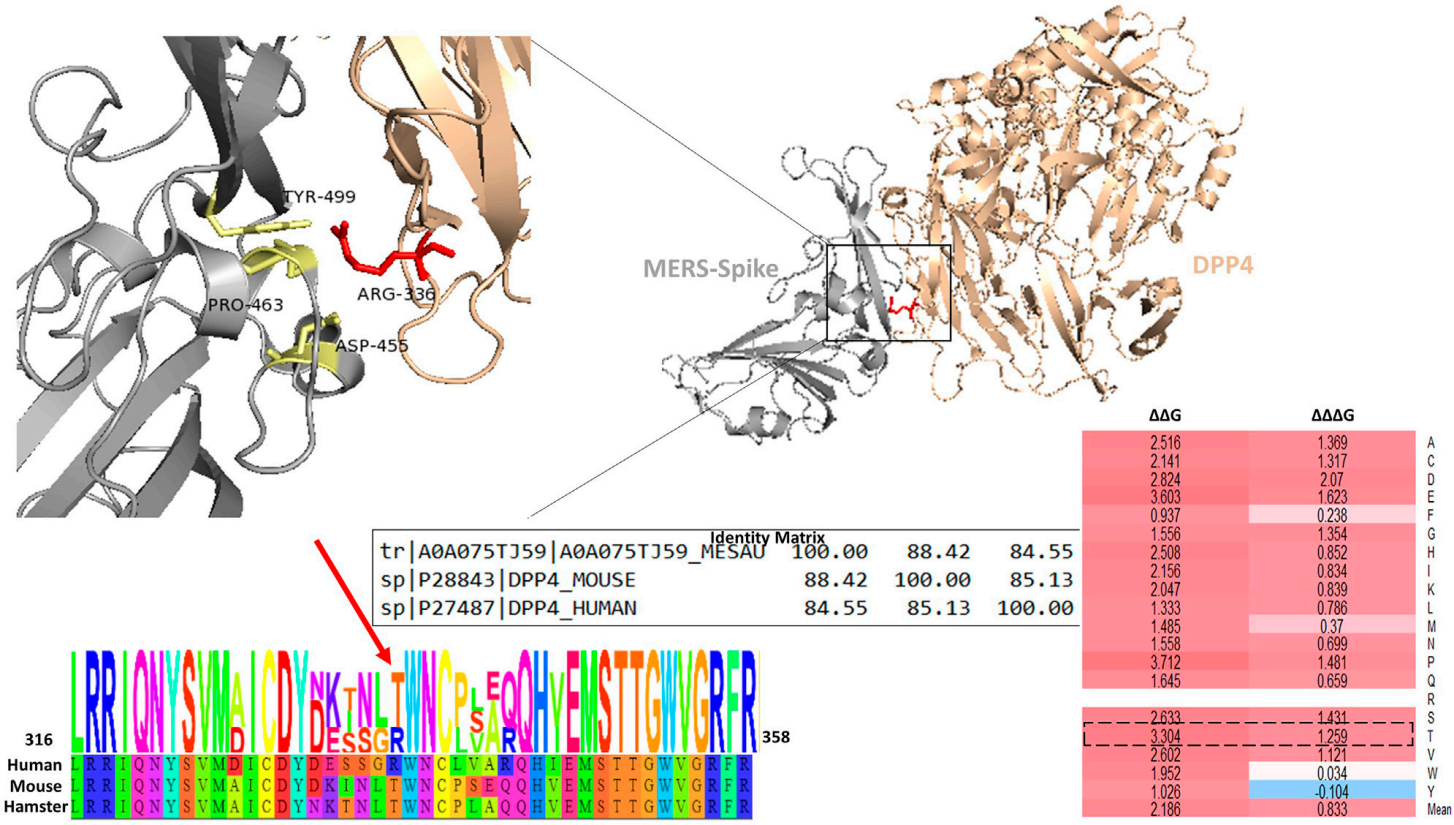
**(A)** Shows the R336T mutation in the interface of the MERS-CoV S RBD and the DPP4 receptor. The identity matrix for the hamster, mouse, and human DPP4 receptors. **(B)** The sequence logo for human, mouse, and hamster is displayed for the sequence surrounding the R336T mutation. **(C)** Is a heatmap showing the ΔΔG and ΔΔΔG values for mutations generated in the position of the R336 residue.

There appears to be no appreciable difference in the overall stability heatmap between the MER-S protein RBD and SARS-CoV-2 external and core subdomains (Figure 8). And the overall distribution of stabilizing and destabilizing mutations appears to be similar as well. The overall percentages of stabilizing and destabilizing mutations are similar between the two subdomains as well. Further examination of the effect of mutagenesis on the components of the RBD subdomains, however, reveals some differences.

Though 60% of mutations generated in element1 of the external subdomains of the MERS-CoVand SARS-CoV-2 S protein RBD result in highly destabilizing effects on free folding energy, the percentage of moderately destabilizing mutations in each protein are 35 and 17%, respectively; additionally, only 4% of mutations are predicted to be neutral in MERS-CoV compared to 18% in SARS-CoV-2. Within element two there is a greater percentage of positions within the SARS-CoV-2 S protein that is predicted to be neutral (42%) when compared to the MERS-S protein (22%), while the percentage of moderately stabilizing mutations is similar between 8 and 9% respectively.

## Discussion

In our study, we found that there was a significant structural similarity in S RBD regions (MERS-CoV v. s. SARS-CoV: RMSD = 1.834 Å, 204 to 195 residues; MERS-CoV v. s. SARS-CoV-2: RMSD = 1.818 Å, 204 to 174 residues). However, the multiple sequence alignment of S RBD regions shows that MERS-CoV has low sequence similarity with SARS-CoV (19.10% identity) and SARS-CoV-2 (17.99% identity). There seem to be no significant differences in the overall heatmaps for the MERS-CoV, SARS-CoV, and SARS-CoV-2 S proteins. In addition to the aforementioned similarities, there is a similar pattern of predicted effects on the protein stability for MERS-CoV, SARS-CoV-2, and SARS-CoV S proteins. Most of the mutations we generated in the these three full-length S proteins and RBD regions were predicted to have destabilizing effects, ranging from 24 to 37.4% (Sobitan et al., 2021; Teng et al., 2021). Mutations predicted to have a neutral effect were also similar between MERS-CoV, SARS-CoV, and SARS-CoV-2 S proteins (25.4–30%). The percentages of stabilizing mutations generated in the three severely pathogenic coronaviruses ranged from 7.4 to 14% (Sobitan et al., 2021; Teng et al., 2021). We found a similar distribution of free folding energy predictions for the RBD region and for the full-length S protein of MERS-CoV. We identified several top positions in the MERS-CoV RBD region where mutagenesis resulted in highly stabilizing or highly destabilizing changes to overall protein stability. Three of the top destabilizing residues, N468, C503, and C526, were also found to close the interaction sites of the MERS-CoV S protein and DPP4. The mutations at these residues may cause geometrical changes of neighbor interaction sites and affect interface stability. In addition, we found that mutagenesis in the six residues (C383, C407, C425, C478, C437, C585) form disulfide bridges that help stabilize the core subdomain could result in destabilization of the MERS-CoV S protein. Protein stability changes to the S protein could affect protein function, and residues that affect protein stability could be targets of drugs and interventions. Further exploration of these comparisons may yield information that could be utilized to generate vaccines or treatments effective against β-CoVs.

We also identified residues that are key to the binding affinity of the MERS-CoV S protein for the DPP4 receptor. It is worth mentioning that the S557 residue is in an equivalent position to the N501 residue in the SARS-CoV-2 virus, based on pairwise sequence alignment. The N501Y mutation has been demonstrated to be important in the increased transmissibility of the B.1.1.7 variant, also known as the Alpha or United Kingdom variant (Liu et al., 2022). Based on pairwise sequence analysis, another residue at the interface of MERS-CoV and DPP4, P463, is in the equivalent position of K417 in the SARS-CoV-2 S protein. The K417N is among several mutations found in the B.1.351 lineage and has been reported to play a role in the immune escape of SARS-CoV-2 (Harvey et al., 2021). A recent investigation showed that plasma from vaccinated study participants was less effective in neutralizing pseudoviruses expressing the K417N variant (Harvey et al., 2021). Understanding mutations that could affect binding affinity could help investigators repurpose drugs and or development new interventions to prevent severe illness.

We found that 62% of all mutations generated in the MERS-CoV S protein RBD region were expected to have destabilizing effects on the overall protein. However, relatively few mutations were predicted to have any effect on the binding affinity of the MERS-CoV S protein. Measuring the binding affinity of single amino acid variations may not fully capture the importance of some viral variants. For example, one investigation of the replication competence of different MERS-CoV clades found that T424I, which is present in the C1.1 clade (Burkina Faso), found replication competence was lower when compared to other strains. The free folding energy prediction indicates that the T424I mutation should moderately increase protein stability (ΔΔG = -0.657). However, this mutation is predicted to have a negligible effect on protein affinity (Zhou et al., 2021). Another study found two viral variations that demonstrated antibody evasion, D510G and I529T (Kleine-Weber et al., 2019). These mutations were predicted to result in small decreases in binding affinity (ΔΔΔG = 0.3629 and 0.4841 kcal/mol, respectively) so minor changes could potentially be important in MERS-CoV infection. However, we did confirm the results of study of DPP4 variants that reduced binding and host entry (Kleine-Weber et al., 2020). In a study of 14 naturally occurring variants of DPP4, the authors found that three variants K267E, K267N, and A291P were associates with reduced binding affinity and reduced host cell entry. In our study all three variants demonstrated moderate to large decreases in the binding affinity for the MERS-CoV receptor (ΔΔΔG: K267E = 2.28, K267N = 1.533, and A291P = 4.158 kcal/mol). Additional variants, in the DPP4, including those that contribute to diseases like diabetes or other disorders, could be important in terms of their affects on binding affinity and viral entry into host cells as well. In addition to demonstrating how missense mutations can affect the MERS-CoV-2 protein stability and its affinity for the DPP4 receptor, we also presented evidence that missense mutations in the DPP4 are also important. One mutation, in particular, the R336T mutation, is particularly important in that this mutation prevents MERS-CoV infection in mice and Syrian hamsters. We demonstrated that this mutation decreases both protein stability and binding affinity for the MERS-CoV S protein. This result further demonstrates the importance of missense mutations in determining protein binding affinity and further illustrates the importance of determining suitable model organisms for conducting β-CoVs.

It is imperative to understand the role of missense mutations both in the evolution of coronaviruses and in the host receptors they exploit. Global pandemics resulting from the spread of novel coronaviruses are a major public health concern due to the risk of mortality and organ damage, as well as the costs associated with treating patients and the burden placed on healthcare services. For example, in 2020, healthcare expenditures in the United States totaled 4.1 trillion dollars, representing an increase of 9.7% (Hartman et al., 2021). Additionally, these viruses have been associated with the development of neurological and neuropsychiatric symptoms associated with long-covid (Montani et al., 2022). Previous work on coronaviruses has shown that the transmissibility of viruses such as SARS-CoV-2 may relate to their ability to replicate in the upper airway. Meanwhile, the fatality of MERS-CoV and similar viruses may relate to its ability to replicate and cause damage within the lower airway (Vu and Menachery, 2021). The transmission, replication competence, protein-protein interaction, and host response related to coronavirus infection all depend on a protein’s ability to function. Therefore, understanding how missense mutations affect protein stability and protein affinity is important, particularly in the development of broadly neutralizing antibodies based therapies to combat coronavirus infection. Investigation of the MERS-CoV S protein mutations could aid in the development of pan-β-CoV vaccines that can target SARS-CoV-2 for current COVID-19 pandemic as well as the putative MERS-CoV-2 or SARS-CoV-3-like coronavirus for the future pandemics.

In summary, we have used computational saturation mutagenesis to investigate the effect of missense mutation on the protein stability and binding affinity of the MERS-CoV S protein. We found that the energetic landscape of MERS-CoV S protein RBD is such that the introduction of missense mutations on average causes destabilization of the overall protein in residues such as G552 and C503. Though several residues demonstrated increased stability such as S435 and S465. In terms of binding affinity, comparatively fewer residues are affected by missense mutations in the MERS-CoV S protein RDB. However, we identified key residues where mutagenesis had a mean stabilizing (S454 and D537) destabilizing effect (E513 and G538) on the complex formed by the S protein and host cell receptor. We also investigated several viral variants, acquired from the ViPR, and identified several viral variants in the MERS-CoV S protein that could affect protein stability or affinity for the DPP4 receptor. We corroborated previous research on naturally occurring variants associated with reduced binding and viral entry into host cells. We also demonstrated reduced protein stability and binding affinity for the R336T mutation that prevents MERS-CoV from infecting mice and Syrian hamsters. And finally, we highlighted several similarities between the MERS-CoV, SARS-CoV, and SARS-CoV-2 viruses. We hope that our research will aid in the identification of drug and intervention targets as well as contributing to the understanding of the ways that missense mutations can impact the structural stability and function of the S protein.

## Data Availability

The original contributions presented in the study are included in the article/Supplementary Material, further inquiries can be directed to the corresponding author.
