# Supplementary material for: In-silico investigation of systematic missense mutations of middle east respiratory coronavirus spike protein
Source: Front Mol Biosci. 2022 Sep 14;9:933553. doi: 10.3389/fmolb.2022.933553 (PMC9515610; doi:10.3389/fmolb.2022.933553)
Supplement: Supplementary file 1 [file DataSheet1.PDF]

Supplementary Material

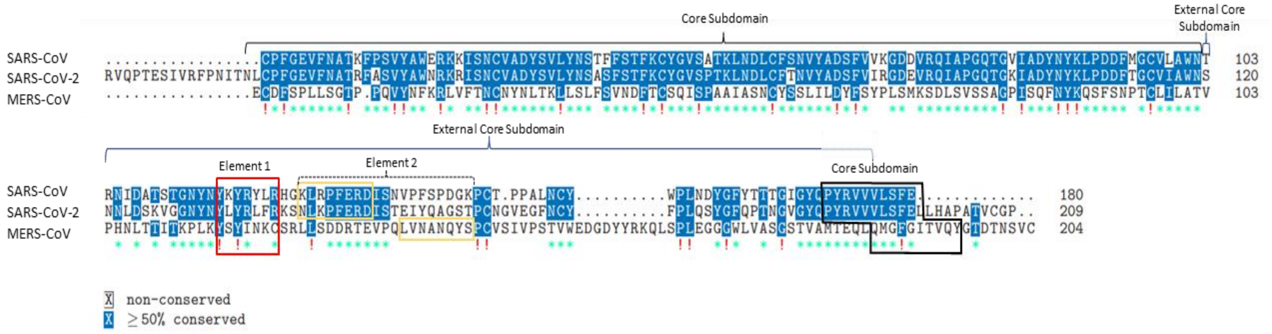

Supplementary Figure 1. Multiple Sequence Alignment of the receptor binding domains of the S-proteins of the SARS-CoV, SARS-CoV-2, and MERS-CoV coronaviruses. The components of the RBD region are emphasized as follows: core subdomain, external core subdomain, element 1, and element 2.

Supplementary Table 1. The effects of viral variants on protein stability, binding affinity and mutation pathogenicity.

	FoldX		SNAP2		
Viral Variant	ΔΔG	ΔΔΔG	Effect	Score	Accuracy
E382V	0.633	0	effect	27	63%
	3.098	0	effect	35	66%
Q395K	1.646	0.0019	effect	15	59%
V396L	2.172	-0.0141	effect	10	59%
Y397P	3.608	0	effect	78	85%
N398E	0.285	0	effect	42	71%
F399L	3.688	0	effect	62	80%
K400E	0.092	0	neutral	-13	57%
R401V	0.187	0	effect	69	80%
L402V	2.117	0	neutral	-7	53%
N406S	0.145	0	effect	41	71%
L414Q	2.543	0	effect	59	75%
S416N	0.106	0	neutral	-27	61%
L417I	0.937	0	effect	13	59%
F418S	6.452	0	effect	50	75%
S419A	-0.514	0	neutral	-60	78%
V420H	2.227	0	effect	52	75%
N421M	0.861	0	effect	51	75%
T424G	1.189	0	effect	50	75%
T424I	-0.657	-1.00E-04	effect	44	71%
C425E	2.962	0	effect	88	91%
S426A	-0.185	0	neutral	-7	53%
Q427R	-0.537	0	effect	33	66%
I428L	0.71	0	neutral	-17	57%
S429D	-2.854	0	neutral	-5	53%
P430S	3.507	0	neutral	-7	53%
A431V	0.213	0	neutral	-42	72%
A432T	-0.364	0	effect	7	53%
A434N	0.735	0	effect	31	66%
S435G	-0.126	0	effect	54	75%
C437T	3.988	0	effect	68	80%
Y438S	5.297	0	effect	78	85%
S439Y	0.002	0	effect	62	80%
S440C	-0.221	0	effect	39	66%
L441A	3.03	0	effect	34	66%
L441V	0.955	0	neutral	-44	72%

I442T	1.907	0	neutral	-66	82%
L443K	1.573	0	effect	63	80%
D444P	0.944	0	effect	84	91%
S451R	-0.867	-0.3479	neutral	-10	53%
M452L	0.31	-0.1223	effect	1	53%
K453E	1.009	-0.0597	effect	43	71%
S454T	0.33	0.0078	neutral	-13	57%
D455N	1.702	1.2273	neutral	-35	66%
L456F	3.131	-0.0024	effect	78	85%
S457M	0.43	-0.0084	effect	1	53%
V458C	0.05	0.0035	neutral	-13	57%
S459T	0.353	0	effect	43	71%
S460G	-0.128	0.3141	effect	52	75%
A461C	0.101	0.0072	neutral	-13	57%
K470T	-0.113	0.021	effect	54	75%
Q471M	-1.7	0	effect	10	59%
S472N	0.027	0	neutral	-40	66%
F473L	0.007	0.0035	effect	38	66%
F473S	0.293	0.0034	effect	20	63%
S474R	0.007	0	effect	9	53%
N475T	0.767	0	effect	7	53%
P476D	3.881	0	effect	40	71%
P485S	2.338	0	neutral	-1	53%
H486F	-0.259	0	neutral	-4	53%
N487D	-1.296	0	neutral	-1	53%
T489S	-0.273	0	neutral	-23	61%
T490A	0.351	0	effect	24	63%
I491V	1.4	0	neutral	-13	57%
T492N	-0.11	0	effect	44	71%
K493N	0.605	0	effect	51	75%
P494G	2.382	0	effect	20	63%
L495M	0.09	-0.0026	neutral	-31	66%
K496S	-0.06	0.0435	neutral	-23	61%
Y497F	1.627	0	effect	73	85%
L506F	10.775	7.39206	effect	75	85%
L517F	-0.215	-0.0386	effect	75	85%
V518C	1.103	0	effect	38	66%
N519L	0.141	0	effect	54	75%
A520S	0.776	0.0013	neutral	-30	61%
N521T	1.792	0	effect	16	59%
Q522E	0.177	0	effect	1	53%
Y523S	2.287	0	effect	82	91%

S524G	0.904	0	effect	57	75%
P525A	2.088	0	effect	12	59%
V527M	-0.448	0	neutral	-50	72%
S528K	-0.592	-1.00E-04	neutral	-3	53%
V530A	2.688	-0.0217	neutral	-29	61%
V530I	0.016	0.0211	neutral	-8	53%
V530L	-0.355	0.0169	neutral	-3	53%
P531V	3.254	0.0425	effect	16	59%
T533Y	0.22	-0.0409	effect	52	75%
D539N	0.453	0.4442	neutral	-14	57%
Y541L	1.858	-0.0634	effect	48	71%
R542L	1.314	0.2939	effect	70	85%
K543R	0.453	0	neutral	-77	87%
L545V	1.607	0.0166	effect	23	63%
P547A	1.468	0	neutral	-24	61%
L548V	0.524	0	effect	19	59%
W553R	3.235	1.5675	effect	84	91%
M569T	3.877	0	effect	69	80%
G570H	18.212	0	effect	81	91%
F571T	4.83	0	effect	74	85%
I573T	2.526	0	effect	60	80%
V575S	3.221	0	effect	79	85%
Q576V	-0.092	0	effect	24	63%
Y577H	0.985	0	effect	65	80%
G578A	2.355	0	effect	64	80%
N582S	0.568	0	neutral	-24	61%
